# Computational analysis of heart rate variability in ASD and ADHD: a systematic review

**DOI:** 10.3389/fncom.2026.1911271

**Published:** 2026-08-13

**Authors:** Raul-Alexandru Gorgan, Teodor-Traian Ştefǎnuţ, Dorian Gorgan

**Affiliations:** Department of Computer Science, Technical University of Cluj-Napoca, Cluj-Napoca, Romania

**Keywords:** ADHD, autism spectrum disorder, autonomic nervous system, computational marker, heart rate variability, neurodevelopmental disorders, nonlinear dynamics, systematic review

## Abstract

Heart rate variability (HRV) represents a rich physiological signal that captures the computational dynamics of autonomic regulation. Emerging evidence suggests that atypical autonomic control contributes to the neurocognitive phenotype of neurodevelopmental disorders (NDD), including autism spectrum disorder (ASD) and attention-deficit/hyperactivity disorder (ADHD). However, the characterization of HRV alterations in these populations remains incomplete. This review was motivated by the broader research context of the EMPOWER project, funded through the Horizon Europe program, which investigates technology-supported approaches for children with NDD, including smartwatch-based physiological sensing and machine-learning analysis of heart rate, HRV, skin temperature, and photoplethysmography signals during cognitive and relaxation tasks. The present manuscript does not report EMPOWER empirical data. Instead, it systematically synthesizes previously published studies on HRV in pediatric ASD and ADHD. The review examines how HRV has been acquired, processed, modeled, and interpreted in pediatric NDD research, with particular attention to nonlinear signal properties, computational approaches, and interactions between autonomic regulation and cognitive processes. Following PRISMA guidelines, the review surveyed publications over the past decade and included 24 empirical studies from five major databases. ASD studies most often indicated altered vagally mediated HRV and autonomic reactivity, whereas ADHD studies more often suggested task-dependent changes linked to attentional control. Cross-study comparability was constrained by substantial heterogeneity in preprocessing pipelines, feature extraction procedures, and analytical frameworks. Despite these methodological inconsistencies, current evidence supports HRV as a promising physiological signal marker of autonomic dysregulation in pediatric NDD. Nevertheless, the field requires standardized signal processing protocols and reproducible modeling approaches to elucidate the mechanistic and predictive relevance of HRV in ASD and ADHD.

## 1 Introduction

Neurodevelopmental disorders (NDDs) represent a group of conditions characterized by early-onset impairments in cognitive, behavioral, social, and adaptive functioning, affecting approximately 15%–20% of children worldwide. According to contemporary diagnostic frameworks such as the Diagnostic and Statistical Manual of Mental

Disorders (DSM-5-TR) (American Psychiatric Association, 2022), NDDs encompass autism spectrum disorder (ASD; prevalence 1%–2%), attention-deficit/hyperactivity disorder (ADHD; 7%–9%), intellectual disability, communication disorders, specific learning disorders, and motor disorders. These conditions typically emerge during early childhood and often persist into adolescence and adulthood, influencing academic achievement, emotional regulation, and social functioning (Yang et al., 2022).

The autonomic nervous system (ANS) is essential for regulating cardiovascular, respiratory, and stress-related physiological processes. Cardiac activity is continuously modulated by the interaction between sympathetic and parasympathetic influences, enabling adaptive responses to cognitive, emotional, and environmental demands. Alterations in autonomic regulation have been increasingly implicated in neurodevelopmental conditions (Mulkey and du Plessis, 2019), suggesting that physiological markers of autonomic function may provide useful information about regulatory mechanisms involved in ASD and ADHD.

Heart rate variability (HRV) is a non-invasive measure of autonomic cardiac modulation, quantified as the temporal variation between consecutive R–R intervals (value between the peaks of heart beats) (Malik et al., 1996; Shaffer and Ginsberg, 2017). HRV indices derived from time-domain, frequency-domain, and nonlinear analyses provide information about autonomic balance, regulatory flexibility, and parasympathetic activity. Reduced HRV has been associated with impaired emotional regulation, attentional control, stress responsiveness, and mental workload (Delliaux et al., 2019; Hye-Geum et al., 2018; Raza et al., 2024). In pediatric populations, HRV is particularly relevant because autonomic regulation undergoes significant developmental changes throughout childhood and adolescence, making HRV assessment a quantitative approach for evaluating regulatory function in children with neurodevelopmental disorders (Gasior et al., 2018; Harteveld et al., 2021; Finley and Nugent, 1995).

From a computational neuroscience perspective, HRV can be considered a dynamic physiological signal that reflects interactions between autonomic control, cognitive state, and behavioral regulation. Previous work has shown that physiological signals such as electrocardiography (ECG), electrodermal activity (EDA), respiration, and related HRV-derived features can be processed through feature-extraction and classification pipelines to characterize affective and cognitive responses (Granero et al., 2016). Similarly, multimodal computational approaches in neurological contexts have demonstrated that cardiac and autonomic features may contribute to predictive modeling when integrated with other physiological data streams (Yang et al., 2023). These findings support the interpretation of HRV not only as a clinical physiological measure, but also as a computationally tractable signal for studying autonomic–cognitive interaction.

The computational relevance of HRV is further strengthened by the fact that heart rhythm dynamics are not fully captured by conventional linear statistics alone. Nonlinear and complexity-based approaches, including entropy measures, Poincare plot descriptors, fractal scaling indices, and multiscale analyses, have been proposed to characterize autonomic dynamics across multiple temporal scales (Nardelli et al., 2015; Gao et al., 2015). Such approaches are especially relevant for pediatric NDD research, where autonomic dysregulation may appear as context-dependent changes in variability, flexibility, or signal complexity rather than as uniform alterations in a single HRV parameter.

Despite the growing interest in autonomic functioning in neurodevelopmental disorders, the existing literature remains methodologically heterogeneous and fragmented. Studies investigating HRV in children with ASD and ADHD vary substantially in sample characteristics, experimental paradigms, recording duration, signal acquisition methods, and HRV analysis approaches (Evans et al., 2013; Weiner et al., 2016). Time-domain, frequency-domain, and nonlinear indices are not consistently reported across studies, and preprocessing procedures are often insufficiently detailed, limiting comparability and reproducibility (Brozat et al., 2025).

Furthermore, HRV has been examined in diverse contexts, including resting-state conditions, cognitive tasks, socio-emotional paradigms, sleep monitoring, and intervention-related settings (Michels et al., 2013; Speer et al., 2024). While this diversity reflects the broad applicability of HRV as a physiological and computational marker, it also complicates the interpretation of findings and the identification of consistent autonomic patterns in pediatric NDD populations. Differences in task design, signal quality, artifact correction, feature extraction, and analytical strategy may strongly influence the reported HRV outcomes.

Currently, no comprehensive synthesis has systematically evaluated how HRV has been acquired, processed, analyzed, and interpreted in children with neurodevelopmental disorders over the past decade (Vidal-Zaborski et al., 2026). A structured review of methodological practices, experimental contexts, HRV feature domains, and computational analysis approaches is therefore necessary to clarify current evidence, identify methodological gaps, and inform future standardization.

The objective of this systematic review is to synthesize how HRV has been operationalized, acquired, processed, analyzed, and interpreted in pediatric ASD and ADHD research over the past decade. In addition to summarizing reported findings, this review evaluates methodological characteristics across studies, including participant demographics, diagnostic criteria, HRV acquisition methods, preprocessing and analysis pipelines, experimental contexts, and selected HRV features. By identifying patterns and inconsistencies in measurement and reporting practices, the review aims to highlight methodological gaps that limit comparability and reproducibility, and to support more standardized use of HRV as a physiological signal marker in pediatric neurodevelopmental research.

This systematic review was conducted within the context of the EMPOWER project, funded through the Horizon Europe program, which aims to develop an innovative learning platform to support children with NDD—including ASD and ADHD—in reducing emotional and behavioral difficulties. The project demonstrated the feasibility of an integrated system combining smartwatch-based physiological sensing, algorithmic analysis, and machine-learning techniques to collect and analyze heart rate, heart rate variability, skin temperature, and photoplethysmography signals during alternating periods of cognitive engagement and relaxation.

Therefore, this review is guided by the following research questions:

- How has HRV been measured, processed, and analyzed in children with neurodevelopmental disorders?
- Which HRV features are most frequently reported in pediatric NDD populations?
- In what experimental and cognitive contexts has HRV been assessed?
- What signal-processing, feature-extraction, and computational analysis approaches have been used to characterize HRV in pediatric ASD and ADHD?
- What methodological limitations and sources of heterogeneity are present across studies?

## 2 Theoretical and computational background

### 2.1 HRV as a signal of autonomic regulation

Heart rate variability (HRV) refers to the physiological variation in the time intervals between consecutive heartbeats, typically quantified from fluctuations between successive R–R intervals derived from ECG recordings or pulse-related intervals obtained from photoplethysmography (PPG). Rather than representing random irregularity, HRV reflects the dynamic modulation of cardiac rhythm by the autonomic nervous system, particularly the interaction between sympathetic and parasympathetic influences on the sinoatrial node (Malik et al., 1996; Shaffer and Ginsberg, 2017).

From a physiological perspective, HRV provides a non-invasive window into autonomic regulation and cardiac adaptability. Higher short-term variability is commonly interpreted as reflecting greater regulatory flexibility, and reduced variability has been associated with stress, reduced vagal modulation, and impaired adaptive capacity (Hye-Geum et al., 2018; Shaffer and Ginsberg, 2017). In children and adolescents, HRV interpretation must also consider developmental maturation of the autonomic nervous system, because age-related changes in cardiac autonomic control can influence baseline HRV levels and responses to environmental or cognitive demands (Finley and Nugent, 1995; Gasior et al., 2018; Harteveld et al., 2021).

In neurodevelopmental research, HRV is relevant because autonomic regulation is closely connected to emotional, attentional, and behavioral control. Altered autonomic modulation may therefore provide physiological information about regulatory difficulties observed in autism spectrum disorder (ASD) and attention-deficit/hyperactivity disorder (ADHD). However, HRV should not be interpreted as a direct diagnostic marker by itself. Instead, it is better understood as a physiological signal that can contribute to the characterization of autonomic regulation when analyzed together with experimental context, participant characteristics, and signal-processing methodology.

### 2.2 Linear and nonlinear HRV features

HRV can be quantified using time-domain, frequency-domain, and nonlinear analytical approaches, each capturing different aspects of cardiac autonomic modulation (Malik et al., 1996; Shaffer and Ginsberg, 2017). From a computational perspective, these measures can be considered features extracted from an interbeat-interval time series, with each feature domain emphasizing a different property of the signal.

Time-domain features quantify the magnitude of variability in successive R–R intervals. Commonly reported indices include the mean R–R interval, the standard deviation of normal-to-normal intervals (SDNN), the root mean square of successive differences (RMSSD), and the percentage of adjacent intervals differing by more than 50 ms (pNN50). Among these, RMSSD and pNN50 are frequently used as indicators of short-term vagally mediated variability, whereas SDNN reflects overall variability influenced by multiple regulatory mechanisms (Malik et al., 1996; Shaffer and Ginsberg, 2017).

Frequency-domain features are derived through spectral analysis of the R–R interval series. The high-frequency (HF) band is generally associated with parasympathetic modulation, and the low-frequency (LF) band reflects mixed sympathetic and parasympathetic influences. The LF/HF ratio has historically been interpreted as an index of sympathovagal balance, although this interpretation remains debated (Malik et al., 1996; Billman, 2013). Frequency-domain measures are frequently used to characterize autonomic responses to stress, emotional stimulation, and cognitive demands (Taelman et al., 2011).

Nonlinear features aim to capture temporal structure, complexity, and scale-dependent properties of heart rhythm dynamics that may not be fully described by linear statistics. These include Poincare plot descriptors such as SD1 and SD2, entropy-based measures such as Approximate Entropy (ApEn) and Sample Entropy (SampEn), and fractal scaling indices obtained through detrended fluctuation analysis (DFA) (Pincus and Steven, 1991; Goldberger et al., 2002). Such features are particularly relevant when HRV is interpreted as a complex physiological time series rather than a set of isolated descriptive statistics.

Previous computational neuroscience studies support this broader interpretation of autonomic signals. Autonomic nervous system dynamics, including standard and nonlinear HRV-related measures, have been used to characterize psychological dimensions and regulatory states (Nardelli et al., 2015). Multiscale entropy analysis has also been applied to biological signals, including HRV, to quantify complexity across multiple temporal scales (Gao et al., 2015). In addition, HRV, ECG, EDA, respiration, and electroencephalogram (EEG) features have been integrated into feature-extraction and classification pipelines for modeling affective or cognitive responses (Granero et al., 2016). These approaches align with the view of HRV as a computationally tractable signal that can support digital phenotyping and physiological modeling when acquisition, preprocessing, and feature extraction are sufficiently standardized.

### 2.3 Autonomic–cognitive regulation in ASD and ADHD

HRV has been increasingly linked to higher-order cognitive processes, particularly those involving attention regulation, executive control, and emotional regulation. Theoretical models of neurovisceral integration propose that cardiac vagal activity reflects functional interactions between prefrontal cortical regions and subcortical autonomic centers (Thayer and Richard, 2000; Thayer et al., 2012). Within this framework, higher resting HRV is generally associated with more efficient top-down regulation and adaptive behavioral control.

Empirical studies have reported associations between reduced HRV and impairments in sustained attention, inhibitory control, and cognitive flexibility (Hansen et al., 2003; Thayer et al., 2012). During cognitively demanding tasks, reductions in parasympathetic indices such as RMSSD or HF power are often interpreted as autonomic withdrawal in response to mental effort (Taelman et al., 2011). Recent computational neuroscience work has also reviewed the use of HRV as a physiological measure of mental workload and cognitive impairment, supporting its relevance for modeling autonomic–cognitive interactions (Raza et al., 2024). Conversely, greater baseline vagal tone has been linked to improved executive functioning and more adaptive emotional regulation (Shaffer and Ginsberg, 2017; Park and Thayer, 2014).

In children with neurodevelopmental disorders, alterations in HRV may therefore reflect atypical autonomic regulation associated with attentional instability, impulsivity, sensory reactivity, or social-cognitive difficulties. In ADHD, HRV may be particularly informative during tasks requiring sustained attention, response inhibition, or performance monitoring. In ASD, HRV may provide information about autonomic responses during sensory, emotional, or social processing. Nevertheless, HRV is not a direct measure of cognitive performance. It should instead be interpreted as an objective physiological correlate of regulatory processes that support attention and executive functioning (Börger et al., 1999).

This distinction is important for computational neuroscience. HRV features may contribute to models of cognitive and affective state, but their interpretation depends on recording context, signal quality, preprocessing decisions, and the selected analytical framework. Multimodal computational studies in neurological contexts have shown that cardiac and autonomic features can support predictive modeling when integrated with other physiological signals (Yang et al., 2023). For pediatric ASD and ADHD research, this suggests that HRV may be most useful not as a standalone biomarker, but as part of reproducible pipelines that combine autonomic signal processing, task context, and clinically relevant behavioral information.

## 3 Materials and methods

This systematic literature review was conducted in accordance with the Preferred Reporting Items for Systematic Reviews and Meta-Analyses (PRISMA) guidelines (Page et al., 2021), which provide a standardized framework for transparent identification, screening, eligibility assessment, and reporting of systematic reviews. The review protocol was defined a priori to ensure consistency in study selection, data extraction, and synthesis.

The scope of the review was restricted to primary empirical studies reporting heart rate variability (HRV) measurements in children or adolescents with neurodevelopmental disorders (NDD). Eligible studies included HRV assessments performed at rest, during experimental or behavioral tasks, during sleep or ambulatory monitoring, or in intervention-related contexts. In line with the computational focus of this review, particular attention was given not only to reported HRV outcomes, but also to signal acquisition methods, preprocessing procedures, feature extraction approaches, and analytical pipelines used to characterize autonomic regulation in pediatric NDD populations. No unpublished EMPOWER data or other primary project-level datasets were included in the screening, extraction, or synthesis.

### 3.1 Search sources and information retrieval

A systematic literature search was conducted across five major electronic databases to identify empirical studies investigating HRV in children and adolescents with NDD. The databases searched were ScienceDirect, IEEE Xplore, PubMed, SpringerLink, and Web of Science.

These databases were selected to provide coverage of biomedical, neuroscience, engineering, physiological monitoring, and interdisciplinary research relevant to HRV analysis, pediatric neurodevelopmental disorders, wearable sensing technologies, and computational signal processing. Only studies published in peer-reviewed journals or conference proceedings were considered eligible for inclusion.

In addition to the primary database searches, Google Scholar was used as a supplementary source to identify relevant peer-reviewed studies from regional or interdisciplinary sources that may not have been retrieved through the main databases. Reference lists of eligible articles were also manually screened to identify additional studies potentially missed by the database searches. No automatic alerts or citation-tracking updates were used after the initial search phase.

The search was restricted to articles published in English from January 2015 onward. This time frame was selected to capture recent methodological developments in HRV acquisition, wearable physiological sensing, signal preprocessing, and pediatric neurodevelopmental research.

### 3.2 Search strategy

The search strategy was designed to identify empirical studies examining HRV in pediatric NDD populations. A combination of free-text keywords and Boolean operators was used to maximize sensitivity across the selected databases.

Search terms were organized around three core concepts: physiological measurement, clinical condition, and population. The primary keywords included:

- Physiological terms: “heart rate variability,” HRV.
- Neurodevelopmental disorder terms: “neurodevelopmental disorder”, NDD, “autism,” “autism spectrum disorder,” ASD, “attention deficit hyperactivity disorder,” ADHD.
- Population terms: child, children, adolescent, pediatric.

The complete database-specific search strings, search fields, date limits, language restriction, and database-specific syntax are provided in the Supplementary material to ensure reproducibility. Search syntax was adapted to each database according to its indexing structure and search interface. No restrictions were applied regarding study design, experimental context, HRV acquisition method, or HRV analytical domain during the initial search phase, in order to capture the full methodological diversity of the field.

### 3.3 Eligibility criteria

#### 3.3.1 Inclusion criteria

Studies were eligible for inclusion if they met predefined criteria related to population, physiological measurement, study design, and publication characteristics. The target population consisted of children and adolescents aged 18 years or younger diagnosed with a neurodevelopmental disorder. These included autism spectrum disorder (ASD), attention-deficit/hyperactivity disorder (ADHD), or other NDD categories, when defined using diagnostic criteria or clinical assessment procedures reported in the original studies.

Eligible studies were required to report HRV as a primary or secondary physiological outcome. HRV had to be explicitly derived from cardiac signals, including ECG, Holter ECG recordings, wearable heart-rate monitoring devices, or PPG-based recordings. Studies were required to report at least one established HRV metric or feature, including time-domain, frequency-domain, nonlinear, or composite autonomic indices.

Only primary empirical studies were included. Eligible designs included observational, case-control, experimental, task-based, sleep-monitoring, ambulatory-monitoring, and intervention-related studies, provided that HRV was measured in pediatric NDD participants. Studies published in peer-reviewed journals or full conference proceedings, written in English, and published from January 2015 onward were considered eligible.

#### 3.3.2 Exclusion criteria

Studies were excluded if they did not meet the predefined inclusion criteria or if their focus was not aligned with the objectives of the review. Studies involving adult populations only were excluded, as the review specifically targeted children and adolescents with NDD.

Articles that did not report HRV as a physiological measure were excluded. This included studies that assessed only heart rate, electrodermal activity, electroencephalography, eye tracking, behavioral performance, or other physiological signals without explicit HRV extraction and analysis. Studies in which HRV was mentioned only superficially, without methodological description or quantitative reporting of HRV metrics, were also excluded.

Non-empirical publications were excluded, including systematic reviews, narrative reviews, meta-analyses, editorials, commentaries, study protocols, and conference abstracts without full papers. Studies focusing primarily on caregivers, parents, or clinicians rather than children with NDD were excluded.

Studies focused exclusively on technology development, assistive systems, virtual or augmented reality applications, or treatment platforms were excluded when they did not include HRV analysis. Publications not written in English or published before January 2015 were excluded to maintain consistency with the defined search scope and contemporary HRV research methodology.

### 3.4 Study selection and screening process

The study selection process followed the PRISMA framework (Page et al., 2021). Following the initial database search, all identified records were exported and organized using Zotero. Duplicate records were identified and removed before title and abstract screening.

In the first screening stage, titles and abstracts were reviewed against the predefined eligibility criteria. Screening was performed by the first author and verified by the other authors, with uncertain cases resolved through discussion until consensus was reached. Records were excluded at this stage when they clearly did not involve pediatric populations, did not assess HRV, did not focus on neurodevelopmental disorders, or represented non-empirical publication types. Full-text versions of the remaining articles were then retrieved and assessed for eligibility.

During full-text screening, studies were excluded if they did not report quantitative HRV parameters, did not include children or adolescents with diagnosed NDD, did not provide sufficient methodological information, or failed to meet publication and language criteria. The selection procedure followed four sequential phases: identification, screening, eligibility assessment, and inclusion. The complete selection process, including the number of records identified, screened, excluded, and included, is presented in the PRISMA flow diagram in Section 4.1. Because screening was performed through verification and consensus rather than fully independent duplicate screening, no inter-rater agreement coefficient was calculated.

### 3.5 Data extraction and synthesis

A structured data extraction process was conducted for all studies meeting the eligibility criteria. For each included article, information was extracted using a predefined framework covering study characteristics, participant characteristics, diagnostic group, HRV acquisition method, experimental context, preprocessing procedures, software tools, and reported HRV metrics.

The extracted study-level data included publication year, country of origin, publication type, study design, sample size, participant age range, sex distribution when available, and neurodevelopmental disorder category. Diagnostic criteria or clinical assessment procedures were also recorded when reported.

Physiological and computational-methodological variables included cardiac signal source, acquisition device, recording duration, sampling frequency, preprocessing and artifact correction procedures, HRV analysis software, and feature-extraction approach. Reported HRV metrics were categorized into time-domain, frequency-domain, nonlinear, and composite feature domains.

Studies were further grouped according to disorder category, acquisition modality, experimental context, signal-processing approach, and primary research objective. Experimental contexts included resting-state paradigms, task-based psychophysiology, sleep monitoring, orthostatic or biomarker-enriched profiling, and system- or prototype-oriented studies.

A quantitative meta-analysis was not performed because the included studies were not sufficiently homogeneous in population characteristics, diagnostic groups, recording protocols, acquisition modalities, preprocessing procedures, experimental paradigms, HRV feature sets, and statistical reporting. Studies differ in whether HRV was assessed during rest, cognitive or attentional tasks, sensory or socio-emotional paradigms, sleep, orthostatic testing, or prototype-based monitoring. In addition, many studies reported different HRV outcomes, used different processing pipelines, or did not provide compatible effect-size data for the same metric under comparable conditions. Pooling these studies would therefore risk producing a summary estimate that obscures clinically and methodologically meaningful differences rather than representing a common effect. Instead, a narrative and descriptive synthesis was conducted. Frequencies and percentages were calculated where appropriate to summarize distributions across studies. The synthesis focused on identifying dominant methodological patterns, sources of heterogeneity, underreported processing details, and gaps relevant to the use of HRV as a computational marker of autonomic regulation in pediatric neurodevelopmental disorders. Extracted data and study categorizations were checked by all authors, and any uncertainties regarding study classification, HRV methodology, or reported features were resolved by consensus.

Methodological quality and risk of bias were assessed using a JBI-informed appraisal approach. Because the included studies varied in design and did not all map directly onto a single Joanna Briggs Institute (JBI) checklist, this assessment was used as a structured narrative appraisal rather than as the formal application of a complete validated JBI critical appraisal tool. The appraisal focused on domains relevant to observational and psychophysiological HRV studies, including participant selection, diagnostic characterization, HRV acquisition, preprocessing and artifact correction, confounder reporting, and appropriateness of analysis. The appraisal was used to guide interpretation of the evidence and was not used as an exclusion criterion. Full appraisal domains and study-level ratings are provided in the Supplementary material.

## 4 Results

### 4.1 Study selection

The initial database search identified 1,115 records across IEEE Xplore, PubMed, ScienceDirect, SpringerLink, and Web of Science. An additional 151 records were identified through supplementary Google Scholar searches and manual screening of reference lists. After duplicate removal, 752 records remained for title and abstract screening.

During title and abstract screening, 687 records were excluded because they did not meet the eligibility criteria. The main reasons for exclusion were non-pediatric populations, absence of HRV measures, lack of focus on neurodevelopmental disorders, non-empirical publication type, or experimental medication protocols. Studies investigating the effects of medication on physiological signals were excluded, whereas studies allowing participants' routine prescribed medication were retained when HRV was otherwise assessed in an eligible pediatric NDD population.

**Figure 1:**
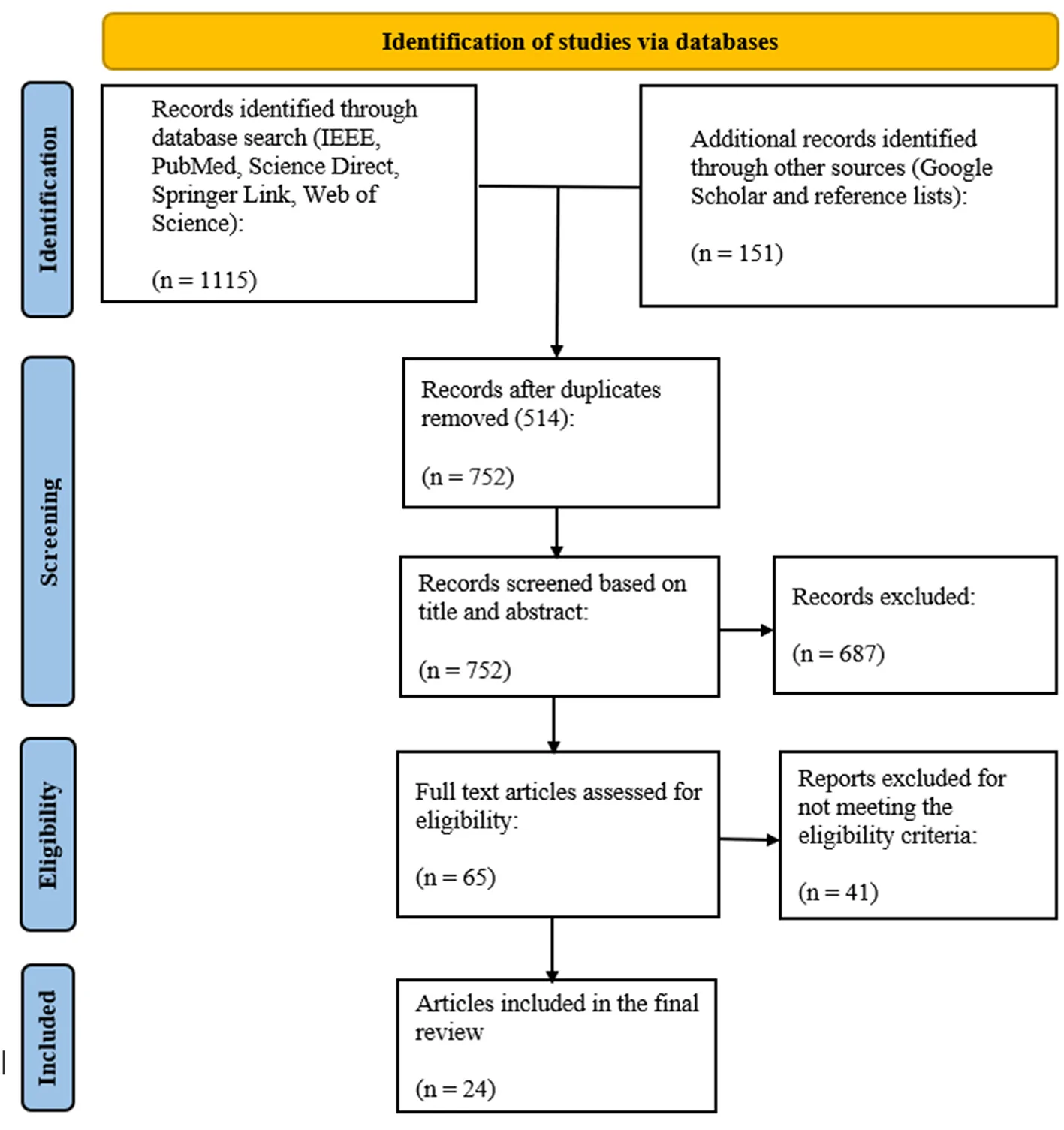
PRISMA flow diagram of the study selection process. The figure shows the number of records identified from the five primary databases and supplementary sources, duplicate removal, title and abstract screening, full-text eligibility assessment, reasons for exclusion, and the final 24 studies included in the qualitative synthesis.

The remaining 65 articles were retrieved for full-text assessment. Following full-text evaluation, 41 studies were excluded because they did not report quantitative HRV metrics, included adult-only samples, were not primary empirical studies, or provided insufficient methodological detail regarding HRV acquisition, preprocessing, feature extraction, or analysis. Ultimately, 24 studies met all eligibility criteria and were included in the qualitative synthesis. The complete study selection process is summarized in the PRISMA flow diagram (Figure 1).

### 4.2 General characteristics of included studies

The final qualitative synthesis included 24 empirical studies published between 2016 and 2026. As shown in Table 1, publication activity was distributed across the last decade, with the highest number of studies published in 2016 and 2021, with four studies each. Three studies were published in 2025, while the remaining years were represented by one or two studies. This distribution indicates a sustained but still relatively limited body of research on HRV in pediatric neurodevelopmental disorders.

**Table 1:** Distribution of included studies by publication year.

Publication year	Number of studies	Percentage
2016	4	16.7%
2017	2	8.3%
2018	2	8.3%
2019	2	8.3%
2020	2	8.3%
2021	4	16.7%
2022	1	4.2%
2023	1	4.2%
2024	2	8.3%
2025	3	12.5%
2026	1	4.2%
Total	24	100%

Most included studies were published as peer-reviewed journal articles. As presented in Table 2, 21 studies were journal articles and three were conference proceedings. Journal publications were mainly located in psychophysiology, neuroscience, developmental disorders, psychiatry, and biomedical research venues, whereas conference papers were more closely related to biomedical engineering, applied sensing, and system-oriented physiological monitoring.

**Table 2:** Distribution of publication types.

Publication type	Number of studies	Percentage
Journal articles	21	87.5%
Conference proceedings	3	12.5%
Total	24	100%

In terms of methodological orientation, the included studies were primarily observational, case-control, or experimental psychophysiology studies. Several studies compared children with ASD or ADHD against typically developing controls (Matsushima et al., 2022; Griffiths et al., 2017; Chiu et al., 2024), while others examined associations between HRV features and clinical, behavioral, cognitive, or physiological variables within clinical groups (Thapa et al., 2021; Kehm et al., 2025; Tonhajzerova et al., 2021). A smaller subset of studies adopted applied or system-oriented designs, integrating HRV into wearable or multimodal physiological monitoring frameworks (Palma et al., 2017; Joshy et al., 2025).

Overall, the included literature shows that HRV research in pediatric neurodevelopmental disorders is mainly represented in clinical, psychophysiological, and biomedical venues, with a smaller contribution from engineering-oriented studies. This distribution is relevant for the present review because it indicates that HRV has been studied both as a physiological marker of autonomic regulation and, more recently, as a signal-processing target in computational and wearable-sensing contexts. Detailed information regarding experimental paradigms and cognitive contexts is synthesized separately in Section 4.7.

### 4.3 Participant and diagnostic characteristics

Across the 24 included studies, participants consisted exclusively of children and adolescents diagnosed with neurodevelopmental disorders, primarily ASD and ADHD. The distribution of studies by disorder category is presented in Table 3.

**Table 3:** Distribution of neurodevelopmental disorder categories.

Disorder category	Number of studies	Percentage
Autism spectrum disorder (ASD)	17	70.8%
Attention-deficit/hyperactivity disorder (ADHD)	7	29.2%
Total	24	100%

ASD represented the majority of the investigated populations, with 17 studies focusing on children with ASD (Mohd et al., 2020; Matsushima et al., 2016), and seven studies examined children with ADHD (Ribeiro et al., 2024; Yuksel and Ozcan, 2018). This distribution indicates that autonomic regulation and HRV-based physiological markers have been investigated more extensively in ASD than in ADHD within the pediatric NDD literature.

Most studies used case-control designs in which children with ASD or ADHD were compared with typically developing control groups (Matsushima et al., 2022; Bujnakova et al., 2016). These designs enabled direct comparison of autonomic regulation between clinical and neurotypical populations. A smaller subset of studies investigated only clinical populations and focused on associations between HRV features and symptom severity, comorbid symptoms, behavioral characteristics, or task-related physiological responses (Kehm et al., 2025; Thapa et al., 2021).

Sample sizes varied substantially across studies. The smallest study included 20 participants (Mohd et al., 2020), whereas the largest cohort included 229 children with ADHD and 244 typically developing controls (Griffiths et al., 2017). Another large study included 198 children (Bazelmans et al., 2019). Most studies included approximately 30–70 participants, reflecting the practical challenges of collecting high-quality physiological recordings in pediatric clinical populations (Matsushima et al., 2022; Chiu et al., 2024; Robe et al., 2021). A few studies included cohorts of around 100 participants or more (Yuksel and Ozcan, 2018; Kehm et al., 2025), representing relatively larger-scale investigations within the reviewed literature.

The reported age ranges spanned early childhood to late adolescence, with study intervals ranging from 1 to 18 years. Across studies, the central age interval was approximately 6–13 years. The mean lower bound of the study samples was 6.3 ± 2.7 years, while the mean upper bound was 12.9 ± 3.5 years. Most investigations therefore focused on school-aged children and early adolescents, a developmental period in which ASD and ADHD are frequently diagnosed, monitored, and clinically evaluated.

Participant characterization was not uniform across studies. Although age and sex were usually reported, the reporting of diagnostic criteria, comorbidities, medication status, symptom severity, and behavioral phenotype varied. This variability is important for HRV interpretation because participant-level factors can influence baseline autonomic tone, task-related reactivity, and the comparability of extracted HRV features across studies.

Overall, the reviewed literature is largely based on moderately sized cohorts of school-aged children, frequently using case-control comparisons between clinical populations and typically developing peers. The predominance of ASD studies, the smaller number of ADHD investigations, and the variability in participant characterization indicate that participant-level heterogeneity remains a major source of variability when interpreting HRV as a physiological or computational marker in pediatric neurodevelopmental disorders.

### 4.4 HRV acquisition modalities and recording protocols

HRV was derived from several cardiac acquisition modalities, including standard ECG, Holter-based ECG monitoring, wearable heart-rate monitors or chest straps, and optical PPG sensors. Some studies used more than one signal source, therefore, acquisition categories are not mutually exclusive. A summary of the acquisition modalities is presented in Table 4, while detailed study-level acquisition information is provided in Table 5.

**Table 4:** Summary of HRV acquisition methods.

Acquisition category	Number of studies	Percentage
ECG/Holter-based acquisition	17	70.8%
Wearable heart-rate monitor/chest strap	6	25.0%
Optical PPG-based acquisition	3	12.5%

**Table 5:** Detailed HRV acquisition methods.

Signal source	Device/hardware	Software/processing	Technical notes
PPG	Optical PPG device not specified	MATLAB; Slope Sum Function (SSF)	HRV; PPG recorded at 1 kHz sampling rate; 4-min recordings (Mohd et al., 2020).
PPG	MAX30102 heart-rate sensor; ESP32-S3 microcontroller	Processing not reported	Prototype system using HRV, GSR, and accelerometer data; data processed by ESP32-S3 (Joshy et al., 2025).
ECG	Biopac MP150	Artiifact software	HRV and skin conductance; 1 kHz sampling rate; 6-min recordings (Chiu et al., 2024).
Holter ECG	Portable ECG Holter	MindWare Bio Lab Acquisition Software; HRV Analyzing Software	HRV; IBI recorded at 500 Hz sampling rate; 50 Hz noise filter; 5-min recording (Thapa et al., 2021).
ECG	Device not specified	Software not specified	HRV and blood pressure; filtering using Smoothness Priors Detrending; 15-min recordings; artifact-free 5-min R–R segment analyzed (Tonhajzerova et al., 2021).
ECG	PowerLab system; disposable electrodes	Heart Rate Variability Module V7; MATLAB	HRV; R–R intervals recorded at 1 kHz sampling rate; 500 Hz anti-aliasing filter; 1 Hz high-pass filter (Matsushima et al., 2022).
ECG	DiANS PF8 device	Manual correction	EDA and HRV; R–R intervals recorded at 1 kHz sampling rate; FFT applied; 5-min recordings (Bujnakova et al., 2016).
Wearable	Polar RS800	Kubios HRV v2.0; Polar Precision Performance software	HRV; R–R intervals; moderate filtering and visual correction; 15-min recordings (Gonzaga et al., 2021).
ECG	Biopac MP150	Kubios HRV Premium	HRV and EDA; 1000 Hz sampling rate; noise detection using Kubios; 25-min recordings (Kehm et al., 2025).
ECG	PowerLab 16-channel system	HRV Analysis Software v1.1	HRV; 1 kHz sampling rate; 15-min recordings; ectopic-free 5-min segment analyzed (Rukmani et al., 2016).
ECG	Compumedics system	In-house Brain Resource software	HRV; 500 Hz sampling rate; low-pass filter at 100 Hz; 50–60 min recordings (Griffiths et al., 2017).
Wearable; ECG	Polar RS800 thoracic belt; ECG ECT WS 2000	Polar software; Kubios HRV	HRV; R–R intervals; ECG recorded at 1000 Hz sampling rate; nocturnal monitoring using R–R intervals; daytime ECG monitoring; 30-min recordings (Pace et al., 2016).
Holter ECG	Holter ECG monitor	Software not specified	HRV; ambulatory 24-h recordings (Yuksel and Ozcan, 2018).
Wearable	Polar H7	Kubios HRV	HRV; R–R intervals; very low filter used; 10-min recordings (Robe et al., 2021).
ECG; PPG	Medicom 83 system	BioSigBrowser in MATLAB	HRV, EDA, respiration, and skin temperature; ECG and PPG recorded at 1000 Hz sampling rate; 13-min recordings (Ribeiro et al., 2024).
Wearable	Polar H10	Kubios HRV v3.5	HRV; R–R intervals; 5-min recordings (Tarlacı and Topal, 2026).
ECG	PowerLab; disposable electrodes	MATLAB	HRV; 1 kHz sampling rate; 1 Hz high-pass filter; 18-min recordings (Matsushima et al., 2016).
Wearable	Polar RS800CX	HRV analysis software	HRV; 5-min recordings; time-domain, frequency-domain, and nonlinear indices (Kalfiřt et al., 2023).
ECG	Biolab MindWare 3.0.4	HRV 3.0.12; MATLAB R2014a; Kubios 2.2	HRV; 500 Hz sampling rate; resting-state recordings (Bazelmans et al., 2019).
ECG	Biopac MP150	HRV analysis software	HRV; short resting-state recordings; 5-min segments; RMSSD and HF (Lory et al., 2020).
ECG	Medical ECG cardio-monitor	HRV analysis software	HRV; head-up tilt test; 1000 Hz sampling rate; time-domain and frequency-domain indices (Bricout et al., 2018).
ECG	PowerLab 26T ECG	Manual Fast Fourier Transform	HRV; resting recordings; frequency-domain indices; 20–25 min recordings (Bharath et al., 2019).
ECG	BrainAmp ECG	QRS Tool; Kubios HRV 4.1.0	HRV; task-based protocol; 1000 Hz sampling rate; time-domain, frequency-domain, and nonlinear indices (Riquelme et al., 2025).
Wearable	Shimmer device	Custom HRV processing	HRV; 20-min recordings; 200 Hz sampling rate; serious games (Palma et al., 2017).

IBI, interbeat interval; EDA, electrodermal activity; GSR, galvanic skin response; FFT, fast Fourier transform; SSF, slope sum function; PPG, photoplethysmography; ECG, electrocardiography.

ECG-based acquisition was the dominant approach. In total, 17 of the 24 studies (70.8%) used ECG-derived R–R interval recordings as a source for HRV analysis (Chiu et al., 2024; Thapa et al., 2021; Bazelmans et al., 2019), while 16 studies used ECG or Holter ECG as the primary HRV acquisition modality. These recordings included standard short-term ECG, portable ECG systems, and Holter monitoring. ECG-based protocols were typically implemented in laboratory or clinical environments, where controlled recording conditions and higher signal fidelity support reliable R-peak detection and HRV feature extraction.

Wearable heart-rate monitors and chest-strap systems were used as the primary acquisition modality in 6 studies (25.0%). These devices were most commonly from the Polar family, including the Polar RS800, RS800CX, H7, and H10 systems (Gonzaga et al., 2021; Robe et al., 2021; Tarlacı and Topal, 2026; Kalfiřt et al., 2023). Wearable systems provide beat-to-beat interval data suitable for short-term HRV analysis and are particularly relevant for pediatric populations because they reduce participant burden and can support more natural recording conditions. One study also used wearable monitoring for nocturnal home-based HRV assessment in combination with daytime ECG acquisition (Pace et al., 2016).

PPG-based acquisition was less frequent. Three studies (12.5%) used PPG as a cardiac signal source, but only two studies used it as the principal acquisition method. One study derived HRV features from short PPG recordings (Mohd et al., 2020), while another incorporated a MAX30102 optical sensor into a prototype monitoring system that also included galvanic skin response and accelerometer data (Joshy et al., 2025). In addition, one multiparametric study combined ECG, PPG, respiration, electrodermal activity, and skin temperature, illustrating an emerging interest in multimodal physiological assessment (Ribeiro et al., 2024).

Reported hardware varied across studies. Laboratory-grade systems included Biopac MP150 (Kehm et al., 2025; Chiu et al., 2024; Lory et al., 2020), PowerLab systems (Rukmani et al., 2016; Bharath et al., 2019), Compumedics acquisition systems (Griffiths et al., 2017), BrainAmp ECG (Riquelme et al., 2025), and other medical ECG devices. Ambulatory or semi-natural protocols included Holter ECG monitoring (Thapa et al., 2021; Yuksel and Ozcan, 2018), Polar chest-strap systems (Gonzaga et al., 2021; Pace et al., 2016; Robe et al., 2021; Tarlacı and Topal, 2026; Kalfiřt et al., 2023), and Shimmer wearable devices (Palma et al., 2017). These differences indicate that HRV research in pediatric NDD populations spans both controlled psychophysiological recording and more applied wearable-sensing approaches. Additional hardware included the DiANS PF8 device (Bujnakova et al., 2016), the Medicom 83 multiparametric system (Ribeiro et al., 2024), and the MAX30102 optical heart-rate sensor used in a prototype monitoring system (Joshy et al., 2025).

When reported, sampling frequencies typically ranged from 200 Hz to 1000 Hz for ECG or PPG recordings (Palma et al., 2017; Mohd et al., 2020; Riquelme et al., 2025). Recording durations also varied substantially, ranging from short 4–5 min resting-state segments to longer task-based recordings, nocturnal monitoring, and 24-h Holter protocols (Mohd et al., 2020; Pace et al., 2016; Yuksel and Ozcan, 2018). Most studies relied on short-term HRV recordings collected under controlled laboratory or clinical conditions, whereas only a small number implemented home-based, ambulatory, or extended-duration monitoring.

Overall, ECG-based acquisition remains the dominant technical approach for HRV analysis in children with neurodevelopmental disorders. Wearable chest-strap monitors and optical sensors are less frequent but increasingly relevant for child-friendly, natural, and multimodal physiological monitoring. From a computational perspective, this variability in acquisition modality, sampling frequency, recording duration, and recording context is important because it directly affects signal quality, beat detection, artifact sensitivity, and the comparability of downstream HRV features across studies.

### 4.5 Preprocessing, feature extraction, and analysis pipelines

**Figure 2:**
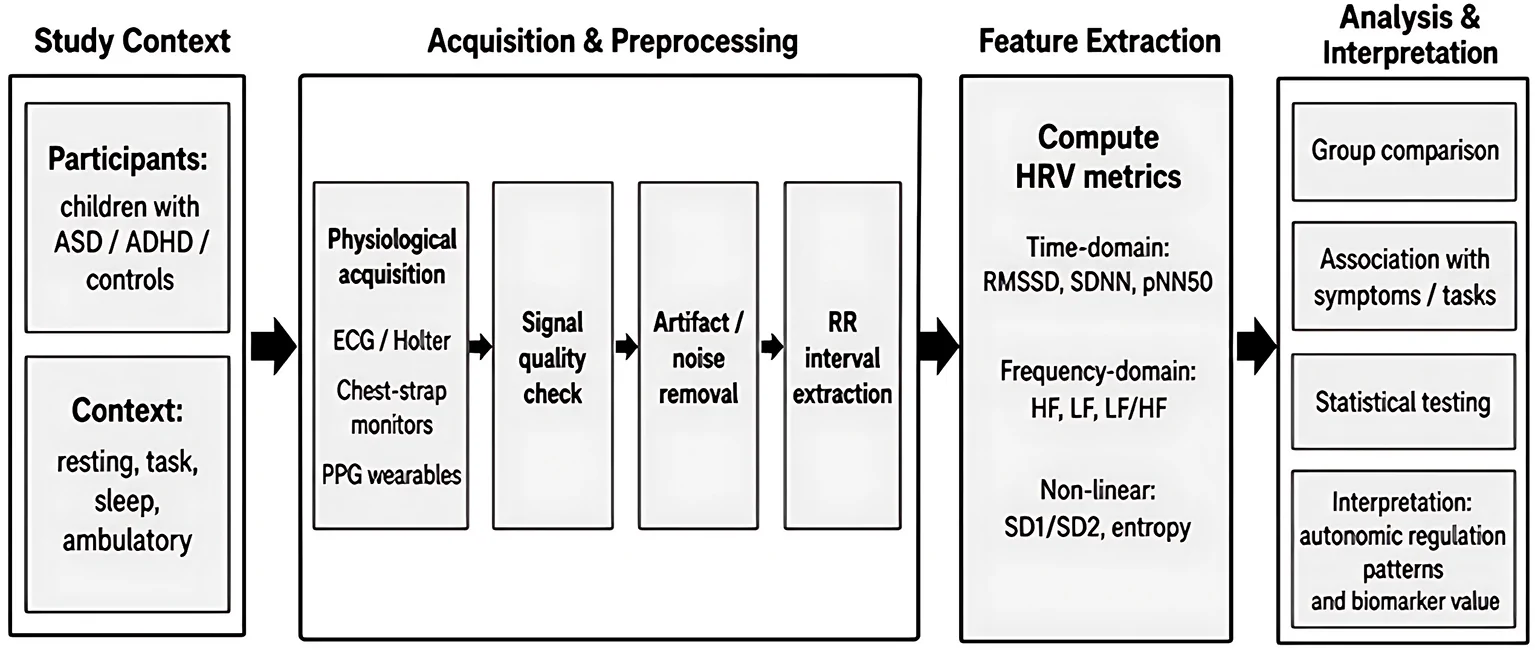
Representative HRV signal-processing pipeline across the reviewed studies. The figure summarizes the main methodological stages, including participant and experimental-context definition, cardiac signal acquisition, signal-quality assessment, preprocessing and artifact correction, R–R interval extraction, HRV feature computation, and statistical or computational interpretation.

Across the included studies, HRV analysis generally followed a common signal-processing workflow, although the implementation and reporting of individual steps varied substantially. As summarized in Figure 2, the reviewed pipelines typically included participant and experimental-context definition, cardiac signal acquisition, signal-quality assessment, artifact or noise correction, R–R interval extraction, HRV feature computation, and final statistical or interpretive analysis. The main sources of methodological variability were acquisition modality, preprocessing quality control, feature-selection strategy, software implementation, and the analytical framework used to relate HRV features to ASD, ADHD, symptom profiles, or task-related responses.

The computational processing of HRV signals was performed using dedicated HRV software, MATLAB-based routines, vendor-specific tools, or custom processing pipelines. Kubios HRV was the most frequently reported software tool and was used for filtering, artifact correction, and extraction of time-domain, frequency-domain, or nonlinear HRV metrics (Gonzaga et al., 2021; Robe et al., 2021; Tarlacı and Topal, 2026; Riquelme et al., 2025). MATLAB-based pipelines were also reported in several studies, either as standalone processing environments or in combination with other software tools (Ribeiro et al., 2024; Bazelmans et al., 2019; Matsushima et al., 2022). Other reported tools included MindWare, HRV Analysis Software, BioSigBrowser, Polar Precision Performance software, and custom or in-house routines (Thapa et al., 2021; Rukmani et al., 2016; Bricout et al., 2018; Griffiths et al., 2017; Palma et al., 2017).

Preprocessing procedures were not uniformly described across studies. Some papers reported R-peak or beat-to-beat interval inspection, filtering, ectopic beat removal, manual correction, or software-based artifact detection (Bujnakova et al., 2016; Gonzaga et al., 2021; Rukmani et al., 2016). Others described specific filtering steps, including noise filtering, high-pass filtering, anti-aliasing filtering, low-pass filtering, or smoothness-priors detrending (Thapa et al., 2021; Matsushima et al., 2022; Griffiths et al., 2017; Tonhajzerova et al., 2021). In several studies, however, artifact handling and preprocessing parameters were only partially specified. This is important because pediatric physiological recordings are vulnerable to motion artifacts, poor electrode or sensor contact, irregular breathing, and task-related movement, all of which can affect beat detection and downstream HRV features.

Feature extraction was dominated by conventional time-domain and frequency-domain metrics. Time-domain features such as RMSSD, SDNN, pNN50, and mean R–R interval were commonly used to characterize short-term variability and overall autonomic modulation. Frequency-domain features, including HF, LF, LF/HF ratio, total power, and very-low-frequency (VLF), were computed using spectral analysis approaches such as fast Fourier transform or software-implemented frequency-domain routines (Bujnakova et al., 2016; Bharath et al., 2019; Riquelme et al., 2025). These linear descriptors formed the core analytical basis of most included studies.

Nonlinear HRV features and composite autonomic indices were reported less frequently but are particularly relevant for computational characterization of autonomic dynamics. Several studies extracted Poincaré plot descriptors, entropy-based measures, detrended fluctuation analysis indices, recurrence-based indices, or composite sympathetic and parasympathetic indices (Mohd et al., 2020; Gonzaga et al., 2021; Kalfiřt et al., 2023; Tarlacı and Topal, 2026; Riquelme et al., 2025). These measures aim to capture signal complexity, temporal structure, fractal scaling, and nonlinear regulatory dynamics that may not be fully represented by conventional linear HRV metrics.

Only a limited number of studies extended HRV analysis toward broader computational or multimodal pipelines. Some studies integrated HRV with EDA, respiration, skin temperature, accelerometry, PPG, or other physiological signals (Ribeiro et al., 2024; Palma et al., 2017; Joshy et al., 2025). These approaches are relevant for digital phenotyping because they treat HRV as one component of a broader physiological feature space. Nevertheless, explicit machine-learning or predictive modeling approaches were rare, and most studies used HRV features for group comparison, association analysis, or task-response characterization.

Overall, HRV processing in pediatric NDD research remains heterogeneous and incompletely standardized. Although several studies used established tools such as Kubios, MATLAB, MindWare, or HRV Analysis Software, reporting of preprocessing choices, artifact correction, feature extraction settings, and software parameters was often incomplete. Future studies should report acquisition parameters, signal-quality criteria, preprocessing steps, artifact handling, feature definitions, spectral-analysis settings, nonlinear-analysis settings, software versions, and statistical or computational modeling procedures in a standardized manner.

### 4.6 Reported HRV features

The included studies reported HRV features across three main analytical domains: time-domain, frequency-domain, and nonlinear or composite measures. Because several studies reported multiple HRV features simultaneously, the frequencies presented in Tables 6–8 are not mutually exclusive. Together, these tables provide an overview of the feature space most commonly used to characterize autonomic regulation in pediatric NDD populations.

**Table 6:** Main time-domain HRV features reported across studies.

Parameter	Studies	Percentage
RMSSD	18	75.0%
SDNN	14	58.3%
pNN50	8	33.3%
Mean RR/RR interval	18	75.0%

RMSSD, root mean square of successive differences between adjacent RR intervals; SDNN, standard deviation of normal-to-normal intervals; pNN50, percentage of successive RR intervals differing by more than 50 ms; RR interval, time interval between consecutive R-peaks in the electrocardiogram.

**Table 7:** Main frequency-domain HRV features reported across studies.

Parameter	Studies	Percentage
HF/HF-HRV / HFA	22	91.7%
LF/LF-HRV	16	66.7%
LF/HF ratio	10	41.7%
Total power (TP)	12	50.0%
VLF	6	25.0%

HF, high-frequency power (typically 0.15–0.40 Hz), commonly associated with parasympathetic (vagal) activity; LF, low-frequency power (0.04–0.15 Hz), reflecting combined sympathetic and parasympathetic influences; LF/HF, ratio between low and high-frequency components, often interpreted as an index of sympathovagal balance; TP, total power of the HRV spectrum; VLF, very-low-frequency power (< 0.04 Hz); HFA, high-frequency activity.

**Table 8:** Nonlinear HRV features and composite autonomic indices reported across studies.

Parameter	Studies	Percentage
SampEn, ApEn, other entropy-based	4	16.7%
SD1/SD2	4	16.7%
DFA α1 / α2	2	8.3%
REC/DET	1	4.2%
SNS/PNS index	2	8.3%

SD1 and SD2, Poincaré plot descriptors representing short-term and long-term variability of RR intervals; DFA, detrended fluctuation analysis, that quantify fractal scaling properties of HRV; REC, recurrence rate and DET, determinism, parameters derived from recurrence quantification analysis (RQA) describing dynamic patterns in the HR time series; SNS, sympathetic nervous system index; PNS, parasympathetic nervous system index, composite measures of autonomic balance derived from multiple HRV parameters; SampEn, sample entropy; ApEn, approximate entropy, used to quantify irregularity in heart rate dynamics.

Time-domain features were widely used to quantify short-term variability and vagally mediated autonomic regulation. RMSSD and mean R–R interval were the dominant time-domain features across the included studies. RMSSD was used in both resting-state protocols and task-based paradigms, indicating its role as a general feature for capturing short-term autonomic modulation (Mohd et al., 2020; Gonzaga et al., 2021; Kehm et al., 2025; Pace et al., 2016; Bazelmans et al., 2019; Riquelme et al., 2025). In ASD-focused studies, RMSSD was commonly interpreted in relation to parasympathetic regulation, emotional processing, or sensory responsiveness (Chiu et al., 2024; Bharath et al., 2019). In ADHD-focused studies, it was more often examined in relation to attentional performance, task-related regulation, or broader autonomic functioning (Griffiths et al., 2017; Robe et al., 2021; Tarlacı and Topal, 2026). Other time-domain features, such as SDNN and pNN50, were used less consistently but provided complementary information about overall HRV and short-term vagal modulation (Yuksel and Ozcan, 2018; Bazelmans et al., 2019; Rukmani et al., 2016; Bharath et al., 2019).

Frequency-domain features represented the most frequently emphasized analytical domain across the included studies. HF and related indices were the dominant spectral measures and were generally interpreted as markers of vagally mediated cardiac regulation. Several ASD studies used HF-related features to examine resting autonomic regulation or responses to emotionally and socially relevant stimuli (Matsushima et al., 2022; Chiu et al., 2024; Bazelmans et al., 2019; Riquelme et al., 2025). In ADHD studies, HF-HRV was more often examined in task-based or performance-related contexts, where it was interpreted as a marker of regulatory flexibility or task-related autonomic modulation (Griffiths et al., 2017; Kehm et al., 2025; Robe et al., 2021). Other spectral features, including LF, LF/HF ratio, total power, and VLF, were used to provide broader descriptions of autonomic balance and spectral variability. However, the physiological interpretation of some frequency-domain measures, particularly LF/HF, remains methodologically debated.

Nonlinear HRV features and composite autonomic indices were reported less frequently than conventional time- and frequency-domain measures. Entropy-based descriptors, Poincaré plot features such as short-term variability (SD1) and long-term variability (SD2), detrended fluctuation analysis (DFA) indices, recurrence-based measures, and composite autonomic indices were used only in a limited subset of studies (Mohd et al., 2020; Kalfiřt et al., 2023; Tarlacı and Topal, 2026; Riquelme et al., 2025). These features are important because they aim to capture signal irregularity, temporal structure, fractal scaling, nonlinear autonomic dynamics, or multidimensional autonomic balance that may not be fully represented by conventional linear HRV measures. One socio-emotional ASD study applied a broader nonlinear framework including DFA and recurrence-based indices, suggesting that nonlinear features may provide additional information about regulatory dynamics during affective or social processing (Gonzaga et al., 2021). Similarly, the use of sympathetic nervous system/parasympathetic nervous system (SNS/PNS) indices in ADHD profiling reflects an emerging interest in composite measures that summarize autonomic regulation across multiple HRV features (Tarlacı and Topal, 2026). The limited use of these measures indicates that more complex HRV representations remain underexplored in pediatric ASD and ADHD research.

Overall, the reviewed literature shows that HRV analysis in pediatric neurodevelopmental disorders is dominated by conventional linear features, particularly HF-related measures, RMSSD, mean R–R interval, and SDNN. Nonlinear HRV features and composite autonomic indices were reported in a smaller subset of studies, despite their relevance for capturing signal complexity, temporal structure, and regulatory flexibility. This imbalance indicates that current HRV research in ASD and ADHD remains primarily descriptive and physiology-oriented, while computationally richer representations of HRV dynamics remain underused. Greater use of nonlinear, multiscale, and composite HRV features may improve the characterization of autonomic regulation and support more robust computational modeling in future pediatric NDD studies.

### 4.7 Experimental and cognitive contexts

The included studies assessed HRV across a broad range of experimental contexts, reflecting the diverse ways in which autonomic regulation has been investigated in children with neurodevelopmental disorders. Based on the dominant recording paradigm, the 24 included studies were grouped into five major categories: resting cross-sectional studies, task-based psychophysiology studies, sleep monitoring studies, orthostatic/biomarker-enriched autonomic profiling studies, and system/prototype-oriented studies, as summarized in Table 9.

**Table 9:** Distribution of experimental contexts across studies.

Experimental context category	Number of studies	Percentage
Resting cross-sectional	10	41.7%
Task-based psychophysiology	11	45.8%
Sleep monitoring	1	4.2%
Orthostasis/biomarker profiling	1	4.2%
System/prototype study	1	4.2%
Total	24	100%

The included studies assessed HRV across heterogeneous experimental and cognitive contexts, reflecting different ways of modeling autonomic regulation in pediatric neurodevelopmental disorders. Based on the dominant recording paradigm, the 24 included studies were grouped into five categories: resting cross-sectional studies, task-based psychophysiology studies, sleep monitoring studies, orthostatic or biomarker-enriched autonomic profiling studies, and system- or prototype-oriented studies. The distribution of these contexts is summarized in Table 9.

Resting-state and baseline-oriented studies formed a substantial part of the literature (Bazelmans et al., 2019; Lory et al., 2020; Bharath et al., 2019). In these studies, HRV was recorded without an explicit cognitive, sensory, or socio-emotional task, usually to characterize baseline autonomic tone, vagal regulation, or associations with clinical characteristics such as emotion dysregulation, sensory sensitivity, or symptom severity. Several ASD studies used this approach, and some ADHD studies also examined baseline autonomic differences between clinical and typically developing groups (Tonhajzerova et al., 2021; Yuksel and Ozcan, 2018). From a signal-interpretation perspective, these paradigms primarily treat HRV as a tonic marker of autonomic regulation. However, the operational definition of “rest” varied across studies, ranging from quiet seated baselines to passive viewing conditions, which limits direct comparability.

Task-based psychophysiology constituted the largest category of experimental contexts (e.g., Griffiths et al., 2017; Kehm et al., 2025; Riquelme et al., 2025; Palma et al., 2017). These studies used HRV to quantify autonomic reactivity, adaptation, or recovery during experimentally induced demands, and were further grouped into sensory/visual stimulation, socio-emotional or affective, attention/executive-performance, and self-regulation paradigms, as presented in Table 10. Sensory-oriented ASD studies measured autonomic responses to tactile, auditory, or visual stimulation (e.g., Chiu et al., 2024), while socio-emotional studies assessed HRV during facial expression processing or affective stimuli (e.g., Riquelme et al., 2025). In contrast, ADHD studies more frequently used attention-demanding paradigms, including sustained attention tasks, baseline-task-recovery designs, computerized attention tests, performance profiling, and delay-related contexts (e.g., Griffiths et al., 2017; Tarlacı and Topal, 2026). This distribution indicates that ASD research has more often used HRV to investigate sensory and emotional regulation, whereas ADHD research has more often used HRV to characterize attentional control, executive regulation, and task-related autonomic flexibility.

**Table 10:** Breakdown of task-based psychophysiology studies by paradigm type.

Task-based paradigm	Number of studies	Percentage
Sensory/visual stimulation paradigms	3	27.3%
Socio-emotional/affective paradigms	2	18.2%
Attention/executive/performance paradigms	5	45.5%
Self-regulation/delay-of-gratification paradigm	1	9.1%
Total	11	100%

The type of task differed by diagnostic focus. ASD studies more often examined autonomic modulation during sensory, visual, or socio-emotional processing, including responses to tactile or auditory stimulation, visual stimulation, facial expressions, or affective stimuli (Chiu et al., 2024; Riquelme et al., 2025). In contrast, ADHD studies more frequently used attention-demanding or performance-related paradigms, including sustained attention tasks, baseline-task-recovery designs, computerized attention tests, and self-regulation or delay-related contexts (Griffiths et al., 2017; Tarlacı and Topal, 2026). This distinction is important because HRV features extracted during sensory or affective processing may reflect different regulatory mechanisms than HRV features extracted during attention, inhibition, or performance monitoring.

Across the task-based studies, experimental paradigms were not distributed uniformly across diagnostic groups. ASD-focused studies more often examined autonomic modulation during sensory, visual, or socio-emotional processing, including responses to tactile or auditory stimulation, visual stimulation, facial expressions, or affective stimuli (Chiu et al., 2024; Riquelme et al., 2025). ADHD-focused studies more frequently used attention-demanding or performance-related paradigms, including sustained attention tasks, baseline-task-recovery designs, computerized attention tests, and self-regulation or delay-related contexts (Griffiths et al., 2017; Tarlacı and Topal, 2026). This distinction is important for interpreting HRV computationally, because features extracted during sensory or affective processing may reflect autonomic reactivity to environmental or emotional stimuli, whereas features extracted during attention, inhibition, or performance monitoring may reflect cognitive effort, executive regulation, or task-related autonomic flexibility.

A smaller number of studies examined HRV in specialized contexts. One study assessed nocturnal HRV during sleep, extending autonomic assessment beyond waking laboratory paradigms (Pace et al., 2016). Another combined resting assessment with orthostatic challenge and biomarker profiling, enabling the evaluation of autonomic adaptation together with cardiovascular or biochemical measures (Bricout et al., 2018; Bharath et al., 2019). In addition, one prototype-oriented study integrated HRV into a multimodal monitoring system together with electrodermal activity, motion sensing, and emotion-related features (Joshy et al., 2025). These specialized paradigms illustrate a gradual shift from isolated HRV assessment toward broader physiological sensing frameworks.

The JBI-informed appraisal indicated that most studies showed low-to-moderate or moderate overall concern. The most common limitations were incomplete reporting of confounders, preprocessing procedures, artifact correction, and signal-quality control. The appraisal domains and study-level ratings are reported in the Supplementary material.

To summarize the strength of evidence across the main findings, a narrative certainty-of-evidence assessment was conducted using GRADE-informed criteria, as shown in Table 11. Because the included studies were heterogeneous and no quantitative meta-analysis was performed, this assessment should be interpreted as a structured narrative judgment rather than as a formal Grading of Recommendations Assessment, Development and Evaluation (GRADE) evaluation. The assessment did not apply a full GRADE evidence-profile procedure or pooled effect estimates, but considered methodological limitations, consistency, directness, precision, and comparability of the available evidence when rating the main conclusions. Overall, the certainty of evidence was low or very low across the main findings, mainly because of methodological heterogeneity, small or uneven evidence bases, incomplete confounder reporting, and limited comparability of HRV acquisition and processing pipelines.

**Table 11:** Narrative certainty-of-evidence assessment for the main review findings.

Evidence statement	Certainty	Main reasons for rating
ASD is associated with altered vagally mediated HRV, especially in HF-related and RMSSD measures.	Low	Findings were reported across several studies, but certainty was limited by heterogeneity in age, recording context, preprocessing, HRV parameters, and confounder control.
ASD studies suggest altered autonomic reactivity during sensory, visual, socio-emotional, or affective contexts.	Low	Evidence was consistent with atypical task-related regulation, but paradigms differed substantially and sample sizes were often modest.
ADHD-related HRV alterations appear more task-dependent and linked to attentional control or executive demands.	Very low–low	Fewer studies focused on ADHD, and task design, medication reporting, comorbidities, and HRV feature selection varied across studies.
Nonlinear and composite HRV features may provide additional information about autonomic complexity.	Very low	Only a small subset of studies reported nonlinear or composite features, and methods were not standardized across studies.
Wearable and PPG-based HRV acquisition may support child-friendly physiological monitoring in pediatric NDD research.	Very low	These approaches are promising, but evidence was limited by few studies, variable validation, and incomplete reporting of signal quality and artifact handling.

Overall, the included studies show that HRV has been analyzed across both tonic and phasic regulatory contexts. Resting-state paradigms primarily characterize baseline autonomic tone, and task-based paradigms capture dynamic autonomic responses to sensory, emotional, cognitive, or behavioral demands. This distinction is important for computational interpretation because the same HRV feature may represent different physiological processes depending on whether it is extracted during rest, stimulation, cognitive effort, recovery, sleep, or ambulatory monitoring. Consequently, heterogeneity in experimental and cognitive paradigms represents a major barrier to cross-study comparison and to the development of reproducible HRV-based computational models in pediatric ASD and ADHD research.

## 5 Discussion

### 5.1 Principal findings

This systematic literature review synthesized evidence from 24 empirical studies investigating HRV in pediatric neurodevelopmental disorders, with emphasis on ASD and ADHD. Across the reviewed literature, HRV was used as a non-invasive physiological signal for characterizing autonomic regulation, but its computational use remains fragmented because acquisition protocols, preprocessing steps, feature sets, and analytical pipelines were not standardized.

Across the included studies, three main patterns were identified. The literature was concentrated within the last decade, was dominated by ASD-focused studies, and relied primarily on ECG-based HRV acquisition. Wearable chest straps, optical sensors, and multimodal physiological systems were less frequent but increasingly relevant for child-friendly and natural monitoring (Gonzaga et al., 2021; Tarlacı and Topal, 2026; Kalfiřt et al., 2023).

From a signal-processing perspective, the reviewed studies were dominated by conventional time- and frequency-domain HRV features, while nonlinear HRV features and composite autonomic indices were used less frequently. This indicates that pediatric ASD and ADHD research still relies mainly on low-dimensional descriptors, although richer representations may better capture autonomic complexity. Experimental context also varied substantially across resting, cognitive, sensory, socio-emotional, sleep, orthostatic, and prototype-based paradigms. Because HRV is strongly context-dependent, this variability limits direct comparison across studies and complicates the development of robust HRV-based computational models for pediatric NDD populations.

These findings indicate that the current evidence should be interpreted more as a heterogeneous methodological landscape than as a unified body of disorder-specific HRV effects. Although several studies suggest altered autonomic regulation in ASD and ADHD, differences in acquisition modality, preprocessing, feature extraction, experimental context, and confounder reporting limit the extent to which findings can be directly compared or generalized. Therefore, the main contribution of the present synthesis is not only to identify recurring HRV patterns, but also to clarify why current evidence remains difficult to translate into standardized computational or clinical markers.

### 5.2 Autonomic and computational HRV patterns in ASD

Within the included literature, ASD represented the most frequently investigated neurodevelopmental disorder, and several studies suggested atypical autonomic regulation in this population. Alterations were most often reported in parasympathetic-related HRV features, particularly HF-HRV and RMSSD, which were commonly interpreted as indicators of cardiac vagal modulation (Bujnakova et al., 2016; Matsushima et al., 2016; Lory et al., 2020). From a computational perspective, these features were generally used as low-dimensional descriptors of autonomic state, supporting comparisons between ASD and typically developing groups or associations with behavioral and clinical characteristics.

Resting-state and task-based ASD studies suggest that HRV alterations may involve both tonic autonomic differences and phasic regulatory responses to sensory, visual, socio-emotional, or affective demands. These findings are consistent with models linking autonomic regulation to emotional regulation, sensory processing, and social adaptation, but they were not fully uniform across contexts or task phases.

Overall, ASD-related HRV research supports the presence of atypical autonomic regulation, but the evidence remains strongly dependent on recording context, preprocessing transparency, and feature selection. Future ASD studies would benefit from standardized HRV acquisition, clearer reporting of artifact handling, and greater use of nonlinear or multimodal computational features capable of distinguishing baseline autonomic tone from dynamic regulatory responses.

### 5.3 Autonomic and computational HRV patterns in ADHD

Compared with the ASD literature, fewer studies investigated HRV in children with ADHD. The available evidence suggests that HRV alterations in ADHD may be especially relevant during tasks involving sustained attention, cognitive control, or performance monitoring, rather than reflecting a single stable resting-state autonomic profile.

Task-based ADHD studies commonly interpreted HF-HRV and RMSSD as indicators of vagally mediated regulation or task-related regulatory flexibility. Some studies also integrated HRV with comorbid symptom profiles or additional physiological signals, including respiration, EDA, PPG, or skin temperature, supporting the interpretation of HRV as part of a broader physiological feature space.

Some ADHD studies also adopted broader autonomic or multiparametric approaches, examining HRV together with comorbid symptoms or anxiety-related profiles (Robe et al., 2021; Kehm et al., 2025), or integrating HRV with respiration, electrodermal activity, PPG, or skin temperature (Ribeiro et al., 2024). These approaches support the interpretation of HRV as part of a wider physiological feature space rather than as an isolated measure.

Overall, HRV may be most informative in ADHD when interpreted as a marker of task-related regulatory flexibility. However, the small number of ADHD studies and variability in task design, acquisition method, preprocessing, and feature selection limit strong conclusions regarding disorder-specific HRV patterns.

### 5.4 Methodological heterogeneity in HRV signal processing

Across the reviewed studies, the main methodological limitation was the lack of standardization in the HRV signal-processing pipeline. Most studies followed a general workflow involving cardiac signal acquisition, beat or R–R interval detection, preprocessing, segmentation, feature extraction, and statistical interpretation, but the technical implementation of these stages was not consistently reported.

Acquisition methods introduced an important source of variability. ECG-based studies enabled direct R-peak detection, whereas chest-strap monitors provided beat-to-beat intervals and PPG-based systems estimated pulse-related intervals from optical signals. These modalities differ in their sensitivity to electrode contact, motion artifacts, sensor placement, peripheral vascular dynamics, and pulse transit time variability. Therefore, HRV features derived from ECG, wearable, and PPG systems are not fully equivalent unless signal-quality control and beat-detection procedures are explicitly described (Gonzaga et al., 2021; Mohd et al., 2020; Joshy et al., 2025).

Preprocessing and segmentation were also inconsistently reported. Some studies described manual inspection, R-peak correction, ectopic beat removal, filtering, artifact detection, or segment rejection, whereas others only specified the software used for HRV analysis. This is technically important because RMSSD and pNN50 are sensitive to local interval correction, frequency-domain features depend on spectral-estimation and windowing settings, and nonlinear measures can be affected by interpolation, filtering, and removal of irregular segments.

Feature extraction was dominated by conventional time-domain and frequency-domain descriptors, including RMSSD, SDNN, HF, LF, total power, and LF/HF ratio. Nonlinear HRV features and composite autonomic indices, such as Poincaré indices, entropy measures, DFA, recurrence-based features, and SNS/PNS indices, were used less frequently. This indicates that most studies relied on low-dimensional HRV representations, while computational methods designed to capture irregularity, fractal scaling, recurrence structure, and multiscale autonomic dynamics remain underused.

The software layer added further heterogeneity, as studies used Kubios HRV, MATLAB-based routines, MindWare, HRV Analysis Software, BioSigBrowser, vendor-specific tools, and custom scripts (Gonzaga et al., 2021; Ribeiro et al., 2024; Bazelmans et al., 2019). These tools may differ in default filters, artifact-correction algorithms, interpolation methods, spectral analysis, and nonlinear-feature computation. Consequently, identical feature labels, such as RMSSD, HF power, LF/HF ratio, SampEn, or DFA α, may not always correspond to identical computational procedures.

Several participant- and protocol-level confounders also limited cross-study interpretation. Medication status was inconsistently reported or controlled, although psychostimulants, anxiolytics, and other prescribed treatments may influence autonomic regulation and HRV estimates. Respiration was rarely monitored or standardized, despite its direct influence on frequency-domain measures such as HF power. Recording duration, body position, and experimental timing also differed across studies, affecting the comparability of short-term resting, task-based, sleep, and ambulatory HRV features. In addition, age, comorbid anxiety or other clinical symptoms, diagnostic heterogeneity, and differences in preprocessing or artifact correction may have contributed to variability in reported HRV findings. These factors should therefore be reported and, where possible, controlled or statistically adjusted in future pediatric ASD and ADHD studies.

Overall, future studies should report the complete HRV workflow, including signal modality, sampling frequency, beat-detection method, signal-quality criteria, artifact correction, interpolation, filtering, segment length, windowing strategy, spectral-estimation method, nonlinear-feature settings, software version, and statistical or machine-learning approach. Such standardization is necessary for reproducible HRV analysis and for comparable computational modeling of autonomic regulation in pediatric ASD and ADHD.

### 5.5 Implications for computational neuroscience and digital Biomarkers

The findings of this review indicate that HRV may support computational characterization of autonomic regulation in pediatric NDD, but only if future studies adopt more reproducible signal-processing pipelines. A complete HRV workflow should report signal modality, sampling frequency, beat detection, signal-quality criteria, preprocessing, artifact correction, segmentation, feature extraction, software settings, and statistical or machine-learning analysis. This is especially important for ADHD, which remains less explored than ASD and requires larger cohorts with clearer reporting of age, medication status, comorbidities, and task context.

Wearable and multimodal physiological monitoring may be particularly useful for digital biomarker development because these systems allow more natural and longer-duration recordings (Gonzaga et al., 2021; Robe et al., 2021; Tarlacı and Topal, 2026; Kalfiřt et al., 2023). However, their use requires robust motion-artifact handling, signal-quality assessment, and transparent preprocessing, especially in pediatric NDD populations where movement and variable task engagement may affect signal reliability.

Future computational work should also move beyond conventional linear HRV descriptors. Nonlinear, multiscale, recurrence-based, and composite autonomic features may better capture regulatory complexity than RMSSD, HF, or LF/HF alone (Mohd et al., 2020; Joshy et al., 2025; Ribeiro et al., 2024). Combined with behavioral, cognitive, and multimodal physiological data, these features may support more robust HRV-based models of autonomic regulation in ASD and ADHD.

## 6 Limitations

Several limitations should be considered when interpreting this review. Although the search retrieved more than one thousand records, only 24 studies met the eligibility criteria, which limits the generalization of the synthesis and reflects the still limited development of HRV research in pediatric neurodevelopmental disorders.

Methodological heterogeneity was the main limitation of the evidence base. The included studies differed in acquisition modality, recording duration, sampling frequency, preprocessing, artifact correction, segmentation, software tools, and HRV feature sets. Experimental contexts also varied substantially, including resting-state recordings, cognitive tasks, sensory stimulation, socio-emotional paradigms, sleep monitoring, and orthostatic assessment. This variability prevented quantitative meta-analysis because no sufficiently homogeneous subset of studies reported the same HRV metric, under comparable acquisition and experimental conditions, with compatible effect-size information.

Participant-level heterogeneity further affected interpretability. Studies differed in age range, sample size, diagnostic criteria, comorbidities, medication reporting, respiration control, recording duration, preprocessing procedures, and control-group design. Several cohorts were small, which may reduce statistical power and increase variability in reported HRV outcomes. In addition, most studies focused separately on ASD or ADHD, limiting cross-disorder comparisons.

The review was restricted to English-language studies published from 2015 onward. This criterion was used to focus on contemporary HRV acquisition and analysis practices, but it may have excluded relevant earlier or non-English studies.

Publication bias could not be formally evaluated because no quantitative meta-analysis was performed and the included studies did not report sufficiently comparable effect-size data for a common HRV outcome. Nevertheless, publication bias remains possible, as small studies with statistically significant or clinically interesting HRV findings may be more likely to be published than studies with null or inconsistent results. Selective reporting of favorable HRV parameters may also have contributed to an overrepresentation of positive findings. Therefore, the conclusions of this review should be interpreted cautiously.

The risk-of-bias assessment should also be interpreted as a JBI-informed structured narrative appraisal rather than as the formal application of a complete validated JBI checklist. Similarly, the certainty-of-evidence assessment was GRADE-informed and narrative rather than a formal GRADE evaluation, because the evidence base was heterogeneous and no quantitative meta-analysis was conducted.

Finally, the synthesis depended on the level of methodological detail reported in the original articles. Incomplete reporting of preprocessing procedures, artifact handling, feature definitions, spectral-analysis settings, nonlinear-feature parameters, or software versions may affect the reproducibility and comparability of the extracted findings. Despite these limitations, the review provides a structured synthesis of current evidence and identifies technical gaps relevant to future HRV-based computational studies in pediatric NDD populations.

## 7 Conclusion

This systematic literature review synthesized evidence from 24 empirical studies investigating HRV in children and adolescents with neurodevelopmental disorders, with emphasis on ASD and ADHD. The reviewed literature shows that HRV is increasingly used as a non-invasive signal for assessing autonomic regulation, but its use as a computational marker remains limited by heterogeneous acquisition protocols, preprocessing procedures, feature sets, and experimental paradigms.

ECG-based acquisition remained the dominant approach, while wearable chest straps, PPG sensors, and multimodal physiological systems were less frequent but increasingly relevant for child-friendly and naturalistic monitoring. Most studies relied on conventional time-domain and frequency-domain features, particularly RMSSD, SDNN, and HF-related indices. In contrast, nonlinear, recurrence-based, multiscale, and composite autonomic features were less commonly applied, despite their potential value for characterizing complex autonomic dynamics.

The available evidence suggests that children with ASD and ADHD may exhibit atypical autonomic regulation, expressed through altered baseline HRV, task-related reactivity, or context-dependent regulatory patterns. In ASD, HRV was most often linked to parasympathetic regulation, sensory processing, and socio-emotional reactivity. In ADHD, HRV was more frequently interpreted in relation to attentional control, cognitive effort, and task-related regulatory flexibility. However, cross-study interpretation remains limited by differences in participant characteristics, recording context, signal quality control, artifact handling, and feature extraction.

Overall, HRV represents a promising physiological signal for studying autonomic regulation in pediatric neurodevelopmental disorders, but its interpretation remains limited by incomplete and inconsistent reporting of the full signal-processing pipeline. The most immediate gap is the lack of standardized reporting for acquisition parameters, beat detection, signal-quality control, preprocessing, segmentation, artifact correction, feature extraction, software versions, and statistical or machine-learning methods. Because most included studies were observational, task-based, or descriptive rather than externally validated predictive modeling studies, computational neuroscience implications should be interpreted cautiously. Addressing this gap should be a priority before HRV can be used more reliably in reproducible computational models of autonomic regulation in ASD and ADHD. Larger cohorts, longitudinal designs, wearable sensing, multimodal physiological data, and nonlinear HRV analysis may support more reproducible and clinically informative models of autonomic regulation in ASD and ADHD.

## Data Availability

The original contributions presented in the study are included in the article/Supplementary material, further inquiries can be directed to the corresponding author.
