# Supplementary material for: Computational analysis of heart rate variability in ASD and ADHD: a systematic review
Source: Front Comput Neurosci. 2026 Aug 13;20:1911271. doi: 10.3389/fncom.2026.1911271 (PMC13518321; doi:10.3389/fncom.2026.1911271)
Supplement: Supplementary file 1 [file Data_Sheet_1.pdf]

Supplementary Material

1 DATABASE SEARCH STRATEGY

The complete search strategy used for each database is reported below to ensure reproducibility. Searches were conducted in PubMed, ScienceDirect, SpringerLink, IEEE Xplore, and Web of Science. The search was restricted to publications written in English and published from January 2015 onward. Where supported by the database interface, filters were applied to retain research articles or full empirical publications and to exclude reviews, editorials, commentaries, protocols, and other non-empirical publication types. Search terms were applied to the broadest available search fields in each database, including title, abstract, keywords, metadata, and full text when supported by the database interface. Search syntax was adapted to the indexing structure and search interface of each database. No restrictions were applied regarding experimental context, HRV acquisition method, or HRV analytical domain during the initial search phase.

2 DATABASE SEARCH STRINGS

2.1 PubMed

Records retrieved: 123.

Search string:

(“heart rate variability”[tiab] OR HRV[tiab]) AND (children OR child* OR pediatric OR adolescent*) AND (“neurodevelopmental disorders”[mh] OR NDD[tiab] OR ASD[tiab] OR “autism spectrum disorder”[mh] OR autism[tiab] OR ADHD[tiab] OR “attention deficit hyperactivity disorder”[mh])

2.2 ScienceDirect

Records retrieved: 437.

Search string:

(“heart rate variability” OR HRV) AND (children OR child OR pediatric OR adolescent) AND (“neurodevelopmental disorder” OR NDD OR ASD OR autism OR “autism spectrum disorder” OR ADHD OR “attention deficit hyperactivity disorder”)

2.3 SpringerLink

Records retrieved: 263.

Search string:

(“heart rate variability” OR HRV) AND (children OR child* OR pediatric* OR adolescent) AND (“neurodevelopmental disorder*” OR NDD OR ASD OR autism OR “autism spectrum disorder” OR ADHD OR “attention deficit hyperactivity disorder”) [All Fields]

2.4 IEEE Xplore

Records retrieved: 46.

Search string:

("All Metadata":HRV OR "All Metadata":"heart rate variability") AND ("All Metadata":children OR "All Metadata":pediatric OR "All Metadata":child OR "All Metadata":adolescent) AND ("All Metadata":"neurodevelopmental disorder" OR "All Metadata":NDD OR "All Metadata":ASD OR "All Metadata":autism OR "All Metadata":"autism spectrum disorder" OR "All Metadata":ADHD OR "All Metadata":"attention deficit hyperactivity disorder")

2.5 Web of Science

Records retrieved: 246.

Search string:

TS=((HRV OR "heart rate variability") AND (child* OR children OR pediatric OR adolescent*) AND ("neurodevelopmental disorder*" OR NDD OR "autism spectrum disorder*" OR autism OR ASD OR ADHD OR "attention deficit hyperactivity disorder"))

2.6 Google Scholar

Records retrieved: 151.

Search string:

("heart rate variability" OR HRV) AND (children OR child OR pediatric OR adolescent) AND ("neurodevelopmental disorder" OR NDD OR ASD OR autism OR "autism spectrum disorder" OR ADHD OR "attention deficit hyperactivity disorder")

2.7 Manual reference-list screening

Manual screening of reference lists from eligible full-text articles identified 11 additional records. Together with the 140 records identified through Google Scholar, this resulted in 151 supplementary records before duplicate removal.

3 JBI-INFORMED RISK-OF-BIAS ASSESSMENT

Risk of bias was assessed using a short appraisal framework adapted from the Joanna Briggs Institute (JBI) critical appraisal domains for observational studies. The tool was simplified to focus on methodological issues most relevant to pediatric HRV research. Ratings were assigned based on the information reported in each included study, with particular attention to HRV acquisition, preprocessing and artifact correction, confounder reporting, and appropriateness of analysis.

Table S1. JBI-informed risk-of-bias appraisal domains.

Domain	Assessment focus
Participant selection	Clear inclusion criteria, appropriate comparison group, and adequate description of participants.
Diagnostic characterization	Clear reporting of ASD, ADHD, or NDD diagnosis and clinical assessment procedures.
HRV acquisition	Valid cardiac signal source, device description, recording duration, and sampling information.
Preprocessing and artifacts	Reporting of filtering, beat correction, artifact removal, segmentation, and software or algorithm settings.
Confounder reporting	Reporting or control of age, sex, medication, respiration, comorbidities, body position, and task context.
Statistical analysis	Appropriate analysis for the study design and HRV outcomes, with sufficient reporting of results.

Table S2. Summary of JBI-informed risk-of-bias assessment across included studies.

Study	HRV method concern	Confounder concern	Reporting concern	Overall concern
Mohd et al. (2020)	Moderate	Moderate	Moderate	Moderate
Joshy et al. (2025)	High	High	High	High
Chiu et al. (2024)	Low	Moderate	Low	Low–Moderate
Thapa et al. (2021)	Low–Moderate	Moderate	Moderate	Moderate
Tonhajzerova et al. (2021)	Moderate	Moderate	Moderate	Moderate
Matsushima et al. (2022)	Low	Moderate	Low	Low–Moderate
Bujnakova et al. (2016)	Moderate	Moderate	Moderate	Moderate
Gonzaga et al. (2021)	Moderate	Moderate	Low–Moderate	Moderate
Kehm et al. (2025)	Low	Moderate	Low	Low–Moderate
Rukmani et al. (2016)	Low–Moderate	Moderate	Moderate	Moderate
Griffiths et al. (2017)	Low–Moderate	Low–Moderate	Moderate	Low–Moderate
Pace et al. (2016)	Moderate	Moderate	Moderate	Moderate
Yuksel and Ozcan (2018)	Moderate	Moderate	Moderate	Moderate
Robe et al. (2021)	Moderate	Moderate	Low–Moderate	Moderate
Ribeiro et al. (2024)	Low–Moderate	Moderate	Low–Moderate	Moderate
Tarlacı and Topal (2026)	Moderate	Moderate	Low–Moderate	Moderate
Matsushima et al. (2016)	Low	Moderate	Low–Moderate	Low–Moderate
Kalfiřt et al. (2023)	Moderate	Moderate	Moderate	Moderate
Bazelmans et al. (2019)	Low–Moderate	Moderate	Low–Moderate	Low–Moderate
Lory et al. (2020)	Low–Moderate	Moderate	Moderate	Moderate
Bricout et al. (2018)	Low–Moderate	Moderate	Moderate	Moderate
Bharath et al. (2019)	Moderate	Moderate	Moderate	Moderate
Riquelme et al. (2025)	Low	Moderate	Low	Low–Moderate
Palma et al. (2017)	Moderate	Moderate	Moderate–High	Moderate–High
